# Cortical atrophy and hypofibrinogenemia due to *FGG* and *TBCD* mutations in a single family: a case report

**DOI:** 10.1186/s12881-018-0597-6

**Published:** 2018-05-16

**Authors:** Joshi Stephen, Sheela Nampoothiri, K. P. Vinayan, Dhanya Yesodharan, Preetha Remesh, William A. Gahl, May Christine V. Malicdan

**Affiliations:** 1 Section of Human Biochemical Genetics, Medical Genetics Branch, National Human Genome Research Institute, National Institutes of Health, Bethesda, MD USA; 2 Department of Pediatric Genetics, Amrita Institute of Medical Sciences and Research Center, Cochin, Kerala India; 3 Department of Pediatric Neurology, Amrita Institute of Medical Sciences and Research Center, Cochin, Kerala India; 4 Department of Pediatrics and Neonatology, Aster MIMS, Kozhikode, Kerala India; 5 NIH Undiagnosed Diseases Program, National Human Genome Research Institute and the Common Fund, 10C-103 10 Center Drive, Bethesda, MD 20892 USA; 6 Office of the Clinical Director, NHGRI, and the NIH Undiagnosed Diseases Program, Common Fund, Office of the Director, National Institutes of Health, Bethesda, MD USA

**Keywords:** *TBCD*, *FGG*, Exome sequencing, Cerebral atrophy, Hypofibrinogenemia, Blended phenotypes

## Abstract

**Background:**

Blended phenotypes or co-occurrence of independent phenotypically distinct conditions are extremely rare and are due to coincidence of multiple pathogenic mutations, especially due to consanguinity. Hereditary fibrinogen deficiencies result from mutations in the genes *FGA*, *FGB*, and *FGG*, encoding the three different polypeptide chains that comprise fibrinogen. Neurodevelopmental abnormalities have not been associated with fibrinogen deficiencies. In this study, we report an unusual patient with a combination of two independently inherited genetic conditions; fibrinogen deficiency and early onset cortical atrophy.

**Case presentation:**

The study describes a male child from consanguineous family presented with hypofibrinogenemia, diffuse cortical atrophy, microcephaly, hypertonia and axonal motor neuropathy. Through a combination of homozygosity mapping and exome sequencing, we identified bi-allelic pathogenic mutations in two genes: a homozygous novel truncating mutation in *FGG* (c.554del; p.Lys185Argfs*14) and a homozygous missense mutation in *TBCD* (c.1423G > A;p.Ala475Thr). Loss of function mutations in *FGG* have been associated with fibrinogen deficiency, while the c.1423G > A mutation in *TBCD* causes a novel syndrome of neurodegeneration and early onset encephalopathy.

**Conclusions:**

Our study highlights the importance of homozygosity mapping and exome sequencing in molecular prenatal diagnosis, especially when multiple gene mutations are responsible for the phenotype.

**Electronic supplementary material:**

The online version of this article (10.1186/s12881-018-0597-6) contains supplementary material, which is available to authorized users.

## Background

The manifestation of two genetically and phenotypically distinct conditions in a single individual is rare and can be due to the co-occurrence of multiple inherited pathogenic loci. Although it is common on patients from consanguineous families due to higher chance of homozygosity of multiple recessively inherited genes, non-consanguineous families with such conditions have also been reported [1]. Fibrinogen, a glycoprotein synthesized in hepatocytes, functions in the final steps of blood coagulation as a precursor monomer of the fibrin hemostatic plug. Fibrinogen deficiency (Factor I deficiency), is a rare inherited bleeding condition due to bi-allelic mutations in one of the three fibrinogen genes *FGA*, *FGB* and *FGG*; these encode α, β and γ fibrinogen polypeptides, respectively, which are folded together to form the mature fibrinogen hexameric structure [2]. Mutations in the fibrinogen genes either affect the quantity of circulating fibrinogen (as in afibrinogenemia or hypofibrinogenemia) or the quality of fibrinogen (as in dysfibrinogenemia) [2]. Symptoms of fibrinogen deficiency include bleeding of the umbilical cord or GI tract, oral and mucosal bleeding, and isolated intracranial bleeding due to traumatic injury; neurodevelopmental symptoms have not been documented [3, 4].

Microtubules are components of the cellular cytoskeleton and are involved in several cellular processes including the cell cycle, motility and intracellular trafficking. In eukaryotes, microtubules form by polymerization of α-β tubulin heterodimers in a head-to-tail fashion, using GTP hydrolysis as the fuel source [5]. Proper polymerization and folding of tubulin monomers involves a series of molecular chaperones (TBCA-TBCE) that assist the formation of α-β tubulin heterodimer [6]. Microtubule polymerization dynamics is crucial for cells, especially for the cellular differentiation and migration of neurons. A spectrum of neurological disorders have been characterized by abnormal neuronal migration and impaired axon guidance due to mutations in the genes that encode α and β tubulin subtypes [7]. Recently, a group of patients was reported with early onset cortical atrophy, neurodegeneration and microcephaly due to bi-allelic mutations in *TBCD*, a tubulin folding chaperone encoding gene [8–10].

In this study, we present a consanguineous family whose proband presented with hypofibrinogenemia and cortical atrophy. Whole exome sequencing revealed that our proband’s blended phenotype is due to mutations in two unrelated genes from two different loci, *TBCD* and *FGG*.

## Case presentation

**Fig. 1:**
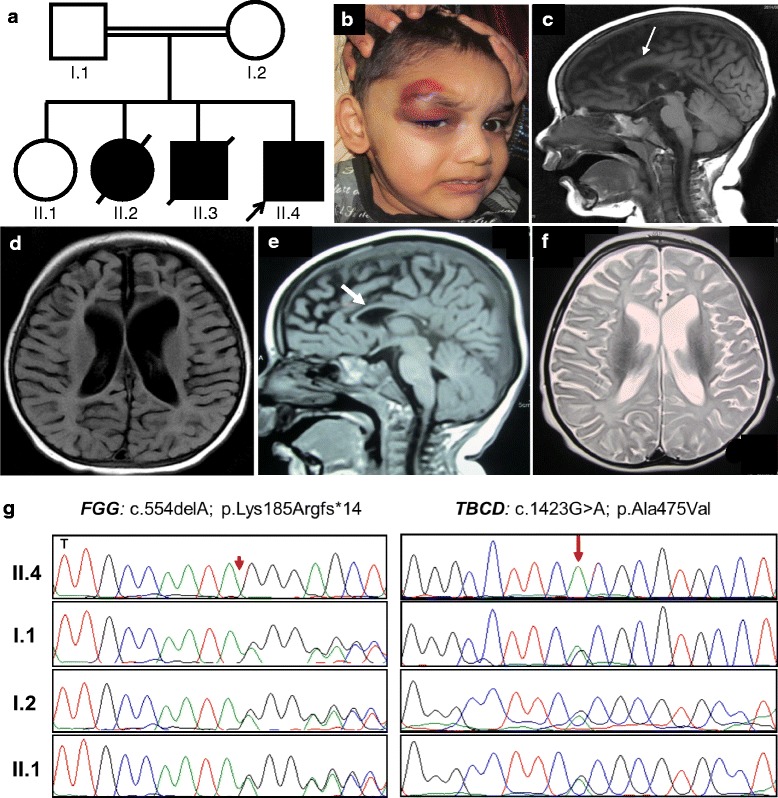
Clinical features and sequencing of the variants. **a** Pedigree shows three affected siblings, the youngest of whom is the proband (arrow) (**b**) Photograph of the proband showing hematoma at the right upper eyelid, microcephaly and deep set eyes. **c-d** MRI brain of the proband (II.4) at 1.5 years of age. T1 sagittal (**c**) and T2 flair coronal (**d**) show significant cerebral cortical atrophy with predominant white matter volume loss along with thinning of the corpus callosum (white arrow). **e-f** Brain MRI of the elder sibling (II.3) at 1 year of age, T1 sagittal (**e**) and T2 axial (**f**). The findings are similar to those of the proband with marked cortical atrophy with white matter volume loss and thinning of corpus callosum (white arrow). **g** Sanger sequencing showing the carrier status of both the variants (*FGG*: c.554delA and *TBCD*: c.1423G > A) in the parents (I.1 and I.2) and unaffected sibling (II.1). The proband (II.4) is homozygous for both the variants. DNA samples from II.2 and II.3 were unavailable

The patient was evaluated at Amrita Institute of Medical Sciences and Research Center, Cochin, Kerala, India. This study was approved by the Institutional Review Board of National Human Genome Research Institute. Informed consent was obtained from the parents to participate in this study. The proband (Fig. 1a, II.4) was a 26-month old male, the fourth child of third degree consanguineous parents from India. He was born full term with a birth weight of 2970 g and had an uneventful antenatal and postnatal period. There were no peripartum or post-partum events suggestive of asphyxia. The proband had an ecchymotic patch at the site of vitamin K injection on the first postnatal day; evaluation for coagulation disorders revealed a prolonged prothrombin time (PT, > 120 s; normal 11- 14 s) and activated partial thromboplastin time (APTT, > 180 s; normal 27-40 s) suggesting reduced fibrinogen levels in the plasma. The prolonged PT, APTT and thrombin time were corrected with normal plasma and a diagnosis of hypo/afibrinogenemia was considered. The infant attained social smile by 2 months and turned over at 4 months. He could sit without support if made to sit by 8 months. He could not grasp objects but could recognize his mother and babble. He lost his ability to sit following the onset of seizures at 8 months. Initial tonic-clonic seizures were followed by flexor spasms. He was treated with sodium valproate and leviterazetam. After 18 months of age, he gradually sat with support and turned over, but did not regain normal head control or pincer grasp. He also had intermittent excessive bleeding from venipuncture sites.

At 24 months of age, the proband's weight was 11 kg (< 5th centile), height 81 cm (< 5th centile) and head circumference of 45.5 cm (< 3rd centile). He had microcephaly, deep set eyes, increased tone in all four limbs with exaggerated deep tendon reflexes and contracture of hamstrings muscles (Fig. 1b). He had a head lag on pulling to sitting position and, on axial suspension, scissored due to excessive axial tone. Visual tracking was absent. Brain MRI at 18 months revealed diffuse cortical atrophy with white matter volume loss and dysgenesis of the posterior corpus callosum (Fig. 1c and d). EEG showed left frontal epileptiform abnormalities during sleep. Nerve conduction velocity showed axonal motor neuropathy affecting bilateral peroneal and ulnar nerves. Fundus evaluation revealed marked temporal disc pallor bilaterally. Visual evoked potentials showed asymmetrically reduced amplitude from the left eye compared to the right eye. Brain stem auditory evoked response (BERA) showed normal latencies of all the waves, with the threshold estimated at 35 dB. Based on his predominant white matter neurodegeneration, enzyme analyses for Krabbe disease and neuronal ceroid lipofuscinosis were performed and found to be normal. At 30 months, the boy developed a huge hematoma involving the right upper eyelid following a trivial fall; the bleeding was controlled with infusion of two units of cryoprecipitate (Fig. 1b).

The Proband had three other siblings. His oldest sibling is a female with normal motor and mental development (II.1). Another sibling, the second child in the family, was a female with prolonged bleeding from the umbilical cord and documented hypofibrinogenemia (II.2). Her birth weight was 3000 g, and there was no birth asphyxia. She had global developmental delay, seizures by 6 months of age, and only partial head control by 15 months. There was no neuroregression. She expired at 15 months of age following an attack of pneumonia. The third child (II.3) in this family was a male with a birth weight of 3650 g. He had normal motor and mental development until 8 months of age and could sit when put in a sitting position. By 8 months, he developed seizures and lost all acquired milestones. He expired at 4 years of age with pneumonia. Brain MRI at 1 year of age showed areas of diffuse cortical atrophy with predominant white matter volume loss and atrophic corpus callosum (Fig. 1e and f). He did not have any bleeding manifestations. The CARE guidelines were followed in reporting this case.

To identify the genetic etiology, SNP genotyping and whole exome sequencing have been performed. SNP genotyping was done on genomic DNA from the proband and parents as previously described [11]. Whole exome sequencing was performed on the proband using the Agilent SureSelect Target Enrichment Kit and the Illumina Hiseq 2000/2500 sequencer (Illumina, Inc., San Diego, CA). Reads were aligned with the human reference genome (hg19; NCBI build 37; Feb. 2009) using Burrows-Wheeler Alignment Tool [12]. Variant calling was performed with GATK [13] and functionally annotated using SnpEff [14]. Given the history of consanguinity in the pedigree, homozygous variants that are in the homozygous areas were filtered based on allele frequency less than 0.01 with no reported healthy homozygotes in online databases, dbSNP, 1000G, ESP6500, ExAC and gnomAD (Additional file 1: Table S1). Pathogenicity was deemed likely if the variant was truncating (splicing or non-sense) or missense and in-frame indels were predicted to be pathogenic using online prediction tools, Polyphen, SIFT, CADD and Mutation Taster. Confirmation and family screening of identified candidates were performed using direct Sanger sequencing (Applied Biosystems).

SNP genotyping identified 14 homozygous regions that are segregating only with the proband (Table 1). One of these regions was in chromosome 4 and included *FGG*, the gene reported to cause fibrinogen deficiency and related to the phenotype of the proband (Table 1). Because the proband’s other siblings presented with hypofibrinogenemia (II.2) and neuroregression (II.3), the possibility of segregation of two recessive disorders in the proband was considered. In search of the gene associated with cortical atrophy, we performed whole exome sequencing on the proband, which revealed 86,697 variants. We focused on the variants in the homozygous areas identified through the SNP array. Autosomal recessive filtering for allele frequencies less than 0.01 and no healthy homozygotes in online databases narrowed the number of variants to four (Additional file 1: Table S2), including *FGG* and *TBCD* (Table 1). The variant in *FGG*, NM_021870.2: c.554del; p. Lys185Argfs*14, is a novel truncating mutation predicted to remove the functional C-terminal region of the protein. The missense mutation in *TBCD,* NM_005993.4: c.1423G > A; p. Ala475Thr, is a known disease-causing mutation (ClinVar, RCV000335816.1; dbSNP, rs775014444). Sanger sequencing confirmed homozygosity of both the mutations; parents and unaffected sibling were carriers (Fig. 1g). DNA of the other affected siblings was unavailable for the analysis.

**Table 1:** Homozygous areas identified in the proband (Fig. 1a-II.4) and the position of candidate genes

Chromosome	Homozygous stretch	Candidate gene
1	142,030,172 - 159,106,629	*GLMP*
3	18,132,514 - 38,613,097	
4	94,947,498 - 122,902,875	*SYNPO2*
4	148,087,853 - 158,539,654	*FGG*
5	78,658,801 - 84,498,637	
7	1 - 3,249,881	
10	71,338,760 - 109,374,231	
12	94,260,616 - 107,839,818	
12	113,571,236 - 118,861,871	
15	20,398,227 - 46,335,050	
17	74,402,197 - 81,195,210	*TBCD*
19	54,610,564 - 59,128,983	
20	44,295,438 - 46,625,603	
22	18,757,589 - 34,101,573	

## Discussion and conclusions

Recent advances in next generation sequencing have greatly advanced molecular diagnosis of monogenic diseases, as well as the identification of cases with blended phenotypes due to multiple gene effects. In this study, we describe the first example of a proband diagnosed with hypofibrinogenemia and a neurodevelopmental disorder associated with homozygous variants in two unrelated genes, *FGG* and *TBCD*. Exome sequencing revealed homozygous variants in four candidate genes including, *GLMP*, *SYNPO2*, *FGG* and *TBCD* (Additional file 1: Table S2). *GLMP* encodes glycosylated lysosomal membrane protein which has role in the metabolic regulation of liver [15]. We excluded this variant since the patient did not have any liver disease. Synaptopodin-2 encoded by the *SYNPO2* gene is implicated in the regulation of cell migration, muscle actin binding and actin bundling [16]. There are no human disease associated with *SYNPO2*, but knockout mice are embryonic lethal and show pre-weaning lethality and abnormal morphology of joints and fingers (IMPC, International Mouse Phenotyping Consortium), hence we excluded this gene. *FGG* and *TBCD* mutations correlated well with the phenotype among the four candidates.

The single base pair deletion in *FGG* identified in our patient predicted a frameshift with protein truncation at position 199 to shift the frame and truncate protein at 199th position. This abolishes the C-terminal region of the fibrinogen gamma chain that contains glutamyl lysine intermolecular cross linking sites essential for the formation of gamma chain dimers [17]. In addition, functional studies demonstrated that mutations affecting the C-terminal region showed either impaired assembly or secretion of the fibrinogen hexamer [18].

TBCD, one of the tubulin five tubulin folding chaperones, is known to be a part of the reversible assembly of the alpha-beta tubulin heterodimer [19]. It is also involved in a wide array of cellular functions including maintenance of microtubular dynamics, assembly and maintenance of the mitotic spindle, cytokinesis, centriolar and ciliary basal body assembly and cell abscission [20]. Recently, bi-allelic mutations in *TBCD* encoding tubulin specific chaperone D were shown to cause neurodegenerative disorders in infants who had initial normal development followed by neuroregression and cortical atrophy [8–10, 21]. The p.Ala475Thr mutation in our patient was shown to be pathogenic in two families of same ethnic descent; affected individuals manifested with severe global developmental delay, dystonia, seizures and brain atrophy [10, 21]. Our patient’s clinical features closely match those of the reported p.Ala475Thr cases (Table 2). The p.Ala475Thr variant has been shown to significantly reduced expression of TBCD due to increased proteosomal degradation, but it had no detectable effect on fibroblast microtubule architecture, translational efficiency and folding or integrity of the protein [10, 21].

**Table 2:** Comparison of clinical presentations of p.Ala475Thr patients

Clinical feature	Edvardson et al., 2016 [10]	Pode-Shakked et al., 2016 [21]	Present case
Ethnicity	Indian Jew (Cochin)	Indian Jew (Cochin)	Indian
Consanguinity	Yes	No	Yes
Number of patients and gender	Two females	One male	One male
Age of onset (months)	3-5	9	8
Initial presentation	Global developmental delay, intractable seizures, brain atrophy, dystonia	Microcephaly, seizures, developmental delay, hypotonia	Seizures, neuroregression, excessive bleeding from venipuncture sites
Microcephaly	Yes	Yes	Yes
Seizures	Yes	Yes	Yes
Optic atrophy	Not available	Yes	Yes
Axial tone	Dystonia	Hypotonia	Hypertonia
Elevated CK	Not available	Not available	Normal
MRI brain	Diffuse cerebral and cerebellar atrophy, thin corpus callosum	Mild to severe cortical atrophy, thin corpus callosum	Diffuse cerebral atrophy, very thin corpus callosum

TBCD defects were initially studied as a possible contributor to the severe microcephaly phenotype in a 7-year-old girl [22], whereby the proband had a combination of a maternally inherited duplication and a missense mutation in *TBCD*, apart from harboring *WDR62* mutations. Our case represents another example in which a *TBCD* mutation contributes a phenotype on top of that attributable to a deleterious *FGG* mutation.

In conclusion, we identified two unrelated homozygous mutations in a single proband, who manifested two distinctive phenotypes associated with the relevant genes. This study shows the importance of performing exome sequencing when patients present with divergent phenotypes.

## Additional file

Additional file 1: **Table S1.** Exome variant filtering strategy of the proband (Fig.1a-II.4). **Table S2.** Final list of candidate genes identified in the proband through exome sequencing. (DOCX 13 kb)
