# Supplementary material for: Cortical atrophy and hypofibrinogenemia due to FGG and TBCD mutations in a single family: a case report
Source: BMC Med Genet. 2018 May 16;19:80. doi: 10.1186/s12881-018-0597-6 (PMC5956920; doi:10.1186/s12881-018-0597-6)
Supplement: Supplementary file 1 — Table S1. Exome variant filtering strategy of the proband (Fig.1a-II.4). Table S2. Final list of candidate genes identified in the proband through exome sequencing. (DOCX 13 kb) [file 12881_2018_597_MOESM1_ESM.docx]

**Table S1**: Exome variant filtering strategy of the proband (Fig.1A-II.4)

| Total number of variants | 86697 |
| --- | --- |
| Homozygous variants | 37731 |
| Homozygous variants that are segregated in the homozygous area | 22801 |
| Exonic and/or splicing variants | 5703 |
| Variants with allele frequency <0.01 in ExAC, 1000Genomes and gnomAD | 36 |
| Indels and missense variants that have no healthy homozygotes identified in publicly available databases and predicted to be pathogenic according to prediction tools such as Mutation Taster, Polyphen-2, SIFT and CADD Phred Score >5 | 4 |

**Table S2**: Final list of candidate genes identified in the proband through exome sequencing

| **Genomic position** | **Gene** | **Variant** | **Allele frequency** |
| --- | --- | --- | --- |
| Chr1:156264285G>T | *GLMP* | NM_001256605.1:c.192C>A; p.Tyr64* | 0.00006091 |
| Chr4:119953030C>T | *SYNPO2* | NM_001128933.2:c.3100C>T; p.Pro1034Ser | 0.00004063 |
| Chr4:155530894del | *FGG* | NM_000509.5:c.554delA; p.Lys185Argfs*14 | Absent in the database |
| Chr17:80828204G>A | *TBCD* | NM_005993.4:c.1423G>A; p.Ala475Thr | 0.00001222 |
